# Electroencephalography-based classification of Alzheimer’s disease spectrum during computer-based cognitive testing

**DOI:** 10.1038/s41598-024-55656-8

**Published:** 2024-03-04

**Authors:** Seul-Kee Kim, Hayom Kim, Sang Hee Kim, Jung Bin Kim, Laehyun Kim

**Affiliations:** 1 Bionics Research Center, Korea Institute of Science and Technology, Seoul, Republic of Korea; 2 Department of Neurology, Korea University Anam Hospital, Korea University College of Medicine, Seoul, Republic of Korea; 3 Department of Brain and Cognitive Engineering, Korea University, Seoul, Republic of Korea; 4 Department of HY-KIST Bio-Convergence, Hanyang University, Seoul, Republic of Korea

**Keywords:** Alzheimer’s disease, Mild cognitive impairment, Subjective cognitive decline, Non-amnestic mild cognitive impairment, Amnestic mild cognitive impairment, Memory-encoding states, Electroencephalography, Alzheimer’s disease spectrum, Computer-based cognitive task, Neuroscience, Diseases, Medical research

## Abstract

Alzheimer’s disease (AD) is a progressive disease leading to cognitive decline, and to prevent it, researchers seek to diagnose mild cognitive impairment (MCI) early. Particularly, non-amnestic MCI (naMCI) is often mistaken for normal aging as the representative symptom of AD, memory decline, is absent. Subjective cognitive decline (SCD), an intermediate step between normal aging and MCI, is crucial for prediction or early detection of MCI, which determines the presence of AD spectrum pathology. We developed a computer-based cognitive task to classify the presence or absence of AD pathology and stage within the AD spectrum, and attempted to perform multi-stage classification through electroencephalography (EEG) during resting and memory encoding state. The resting and memory-encoding states of 58 patients (20 with SCD, 10 with naMCI, 18 with aMCI, and 10 with AD) were measured and classified into four groups. We extracted features that could reflect the phase, spectral, and temporal characteristics of the resting and memory-encoding states. For the classification, we compared nine machine learning models and three deep learning models using Leave-one-subject-out strategy. Significant correlations were found between the existing neurophysiological test scores and performance of our computer-based cognitive task for all cognitive domains. In all models used, the memory-encoding states realized a higher classification performance than resting states. The best model for the 4-class classification was cKNN. The highest accuracy using resting state data was 67.24%, while it was 93.10% using memory encoding state data. This study involving participants with SCD, naMCI, aMCI, and AD focused on early Alzheimer’s diagnosis. The research used EEG data during resting and memory encoding states to classify these groups, demonstrating the significance of cognitive process-related brain waves for diagnosis. The computer-based cognitive task introduced in the study offers a time-efficient alternative to traditional neuropsychological tests, showing a strong correlation with their results and serving as a valuable tool to assess cognitive impairment with reduced bias.

## Introduction

Alzheimer’s disease (AD) is a progressive and irreversible condition in which neurons lose their function and connections over time, resulting in deterioration of cognitive abilities, such as reasoning, memory, and sense of direction^1^. Mild cognitive impairment (MCI) is considered a transitional phase between normal aging and AD, and research suggests that, each year, approximately 8–15% of MCI patients develop AD^2^. Individuals with MCI have minor cognitive impairments that do not significantly interfere with their daily activities; therefore, MCI is often overlooked and misrecognized as a manifestation of normal aging^3^. However, studies indicate that MCI patients progress to AD earlier than healthy individuals of the same age^4^. Therefore, early MCI detection is essential for controlling disease progression and delaying interference with daily activities^1^. Particularly, non-amnestic MCI (naMCI) is often confused with normal aging because it lacks the representative AD symptom, memory decline^5^. Subjective cognitive decline (SCD) is an intermediate step between normal aging and MCI and is crucial for the prediction or early detection of MCI, which determines the presence of AD spectrum pathology^6^. Therefore, developing multi-class classification models for differential diagnosis of SCD, naMCI, aMCI, and AD, will be helpful in demonstrating the presence of AD pathology and its corresponding stage in the AD spectrum, and to develop strategies to prevent progression.

AD diagnosis involves neuropsychological tests, cerebrospinal fluid (CSF) analysis, brain imaging, and electroencephalography (EEG) that evaluate cognitive functions and identify secondary causes for cognitive decline^7^. Neuropsychological tests evaluate cognitive decline indicative of AD and consist of paper-and-pencil and face-to-face tests for various cognitive domains. As neuropsychological tests rely on patients cooperation, they have limited sensitivity and reproducibility. Therefore, objective indicators measuring cognitive functions are required^8,9^. The Amyloid/Tau/Neurodegeneration (ATN) framework, proposed by the National Institute on Aging and Alzheimer’s Association in 2018^10^, is considered the gold standard for diagnosing Alzheimer’s disease. In the ATN framework, the biological state of AD is determined by analyzing three biomarkers (amyloid, tau, and neurodegeneration) obtained from CSF and positron emission tomography (PET) imaging^10^. Although the ATN framework is effective for AD diagnosis, it usually requires lumbar puncture or PET scans, which are expensive, invasive and highly dependent on the clinical infrastructure, severely limiting its applicability.

Other diagnosis tools include imaging techniques such as magnetic resonance imaging and signal photon emission computed tomography, which provide structural and functional information about the brain^11^. Although brain imaging provides valuable information regarding cognitive dysfunction and aids in AD diagnosis, alterations in brain structure are typically observed only in advanced stages of the AD spectrum. Moreover, functional imaging is used for research purposes rather than clinical applications. Additionally, altered findings of functional imaging are not necessarily correlated with structural changes. Therefore, clinical application of neuroimaging techniques for early diagnosis of AD spectrum is limited. There is an increasing effort to develop new approaches for differential diagnosis of the AD spectrum by overcoming the limitations of conventional diagnostic tools^12,13^.

Recently, quantitative analysis of EEG has been considered as an alternative approach for developing objective markers and models for differentiating AD spectrum. EEG is an inexpensive, portable, and non-invasive neurophysiological technique capable of measuring brain activity and detecting neurological diseases through signal processing and analysis theories^14–24^. According to various reports, the classification between MCI and Health Control (HC) is more challenging than the classification between AD and HC. Gallego et al.^16^ classified MCI versus HC and AD versus HC. As a result of linear discriminant analysis (LDA) classification characterized by relative power, the classification accuracy of AD was 97.56% and MCI was 78.33%. Meghdadi et al.^17^ achieved an AUC of 0.85 for AD and only 0.6 for MCI using LDA classification with power spectral density (SD) and coherence. Jiao et al.^18^ also reported higher classification accuracy in HC versus AD (85.8%), than HC versus MCI (79.8%) using LDA.

While most EEG studies identified significant differences between the two specific stages of the AD spectrum and achieved successful binary classifications, only a few studies attempted to develop multi-class classification models. In 2019, Ieracitano et al.^19^ presented a 3-way classification algorithm employing a convolutional neural network approach using the PSD features of the eye-closed state and obtained an accuracy of 83.33%. In 2020, Ieracitano et al.^20^ introduced a multi-layer perceptron classifier utilizing continuous wavelet transform (CWT) and a bispectral (BiS) representation and obtained an 89.22% accuracy for a three-way scheme. Oltu et al.^21^ achieved 93.88% accuracy using a bagged trees classifier employing discrete wavelet transform, PSD and coherence as features for the EEG with eye closed-states for 35 subjects (8 AD, 16 MCI and 11 HC). Most recently, in 2023, Jiao et al.^18^ utilized a large sample size (246 HC, 189 aMCI and 330 AD) and performed triple classification (HC, aMCI, and AD) with 70.2% accuracy.

Most studies have measured data in the resting state (Table 1) and only a few researchers have attempted to diagnose and predict AD using the EEG obtained during cognitive performance state. The 3-way (HC, MCI, and AD) SVM-based classifier proposed by McBride et al.^22^ achieved classification accuracies of 79.2%, 83.3%, and 85.4% for the eyes-closed resting state (EC), eyes-opened resting state (EO) and EC with counting tasks, respectively. Sharma et al.^23,24^ achieved accuracies of 80.10%, 85.09%, and 87.59% in classifying EEG measurements during EO, EC, and a motor speed test (MST) into 3-classes, respectively, suggesting that the MST is the most reliable test for diagnosing dementia. These results suggest that classification of the AD spectrum would be more accurate with using task-derived EEG data than resting-state data.

**Table 1:** Comparison of the method and results in the literature.

Classification	Study	Cohorts	Event	Features	Classifier	Test performance		
						*acc*	*F1*	*AUC*
2 Class	Balbiloni^25^	AD 42/HC 40	EC 5 min	LLC	Statistical analysis	78.10%		
	Bairagi^36^	AD 50/HC 50	EC 15-20 min	Spectral and Wavelet features	SVM	94.00%		
					kNN	92.00%		
	Amezquita^26^	AD 37/MCI 37	EC 35 s	fractality dimension(FD), Hurst exponent(HE)	EPNN	90.30%		
					kNN	89.10%		
					NB	88.70%		
	Ruiz^27^	AD 37/MCI 37/SCD 37	EC 5 min	RP, sample entropy	MLP	77.45%		
					QDA	76.47%		
					LDA	75.49%		
	Meghdadi^17^	AD 26/MCI 53/SCD 55	EC 5 min, EO 5 min	PSD, coherence	LDA	–	–	0.725
	Sharma^23^	AD 15/MCI 16/HC 13	EO 3 min	PSD, Skewness, Kurtosis, Entropy, Fractal Dimension	SVM	78.75%		
			EC 3 min			83.05%		
			FTT 2 min			86.80%		
			CPT 5 min			80.95%		
	AI^15^	VaD 5/MCI 15/HC 15	Memory recall task 1 min (5word)	RP, Permutation Entropy, Fractal Dimension, Wavelet features,	SVM	91.48%		
					ckNN	89.63%		
3 Class	Ieracitano^19^	AD 63/MCI 63/HC 63	EC	PSD	CNN	83.33%	81.17%	0.94
					MLP	56.40%	58.73%	0.8
					SVM	56.84%	56.06%	0.79
					LDA	55.70%	57.76%	0.77
	Ieracitano^20^	AD 63/MCI 63/HC 63	EC	CWT, BiS	MLP	89.22%	80.87%	
					SVM	74.54%	60.09%	
	Oltu^21^	AD 8/MCI 16/HC 11	EC 7 min	DWT, PSD, coherence	Bagged Trees	93.88%		
	McBride^22^	AD 17/MCI 16/HC 15	EO 5 min	spectral and complexity features	SVM	83.30%		
			Counting EC with finger-tapping 10 min			85.40%		
			EC 10 min			79.20%		
	Sharma^24^	AD 16/MCI 16/HC 15	EO 3 min	Spectral kurtosis, skewness, spectral entropy, fractal dimension, PSD of beta, gamma	SVM	80.10%	79.39%	
			EC 3 min			85.09%	84.79%	
			MST with finger-tappling 2 min			87.59%	86.78%	
	Jiao^18^	AD 330/aMCI 189/HC 246	EC 10 min	PSD, Hjorth metrics, Sample entropy, microstate measures	LDA	70.00%	69.40%	
					SVM	70.20%	69.80%	

LLC, Log Linear Connectivity; MLR, Multinomial and Logistic Regression; MLP, Multi Layer Perceptron; PSD, Power Spectral Density; RP, Relative Power. AP, Absolute Power; CWT, Continuous Wavelet Transform; Bis, bispectrum; DWT, Discrete Wavelet Transform; MST, Motor Speed task; FTT, Finger Tapping Test; CPT, Continuous Performance Test; VaD, Vascular Dementia; EC, Eye Closed; EO, Eye Opened.

**Figure 1:**
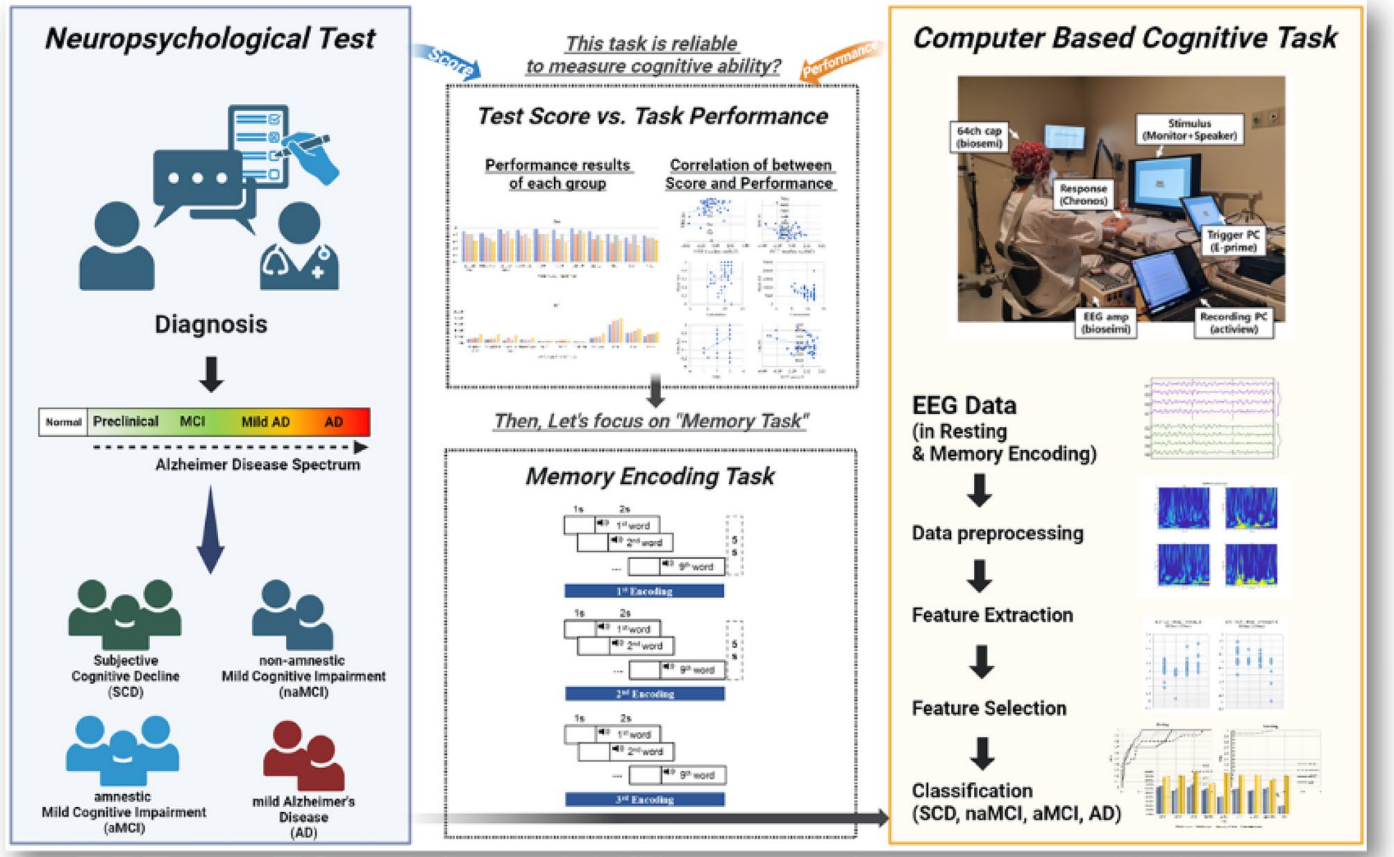
Schematic overview of experiment.

In this study, we used EEG data during “Memory Encoding” session and “Rest with Eye opened” in a computer-based cognitive task to diagnose SCD, naMCI, aMCI, and AD. The purpose of this study is as follows: First, we created a computer-based cognitive task to measure behavioral data and bio-signal (ex. EEG, ECG, EMG, etc.) data for five cognitive areas. It is reliable and faster compared to conventional neuropsychological tests. The presence of biometric data helps prevent inconsistencies in the results due to the subject’s incorrect response rate owing to tension or mistake. Second, based on the previous results^22–24^, we hypothesized that EEG data during the cognitive functioning state is more effective for classification than the resting-state. Therefore, the EEG data during memory encoding reflecting the cognitive activity of memory, which is the fundamental domain for diagnosis of AD spectrum, was compared with that during resting. Through this, we aimed to examine the difference in the EEG pattern among the clinical stages of the AD spectrum, as it could be used for classification. Finally, we applied machine learning (ML) and deep learning (DL) models for classifying the clinical stages of the AD spectrum into four groups (i.e., SCD, naMCI, aMCI, and AD). The overall scheme of this study is presented in Fig. 1.

## Materials and methods

### Participants

The study followed the ethical guidelines of the Declaration of Helsinki and was approved by the local ethics committee of Korea University Anam Hospital (No. 2021AN0266). Informed consent was obtained from all the participants. A total of 60 participants were recruited for this study. Two were excluded because of missing data or dropouts. Table 2 shows the participants’ group, sex, age, years of education, and cognitive test score distributions. The Mini-Mental State Examination (MMSE) was used to assess global neurocognitive function. All participants underwent neuropsychological testing using the Seoul Neuropsychological Screening Battery second edition (SNSB-II)^28^.

**Table 2:** Demographics and clinical data of participants.

	SCD	NaMCI	aMCI	AD
N	20	10	18	10
Gender (M/F)	11/9	4/6	9/9	4/6
Age (Years)	63.42 ± 11.18	69.60 ± 3.47	66.17 ± 8.74	71.20 ± 6.76
Education (Years)	9.33 ± 4.43	6.90 ± 4.50	11.78 ± 3.36	10.00 ± 4.00
MMSE	28.37 ± 1.60	25.30 ± 3.03	26.61 ± 2.58	23.40 ± 2.94
Memory	0.30 ± 0.76	− 0.64 ± 0.40	− 1.87 ± 0.78	− 2.66 ± 1.38
SGDS	4.53 ± 4.20	8.00 ± 4.90	4.89 ± 3.91	6.40 ± 4.15
B-ADL	20.00	18.90 ± 2.39	19.67 ± 0.82	19.44 ± 1.07
K-IADL	0.06 ± 0.09	0.18 ± 0.13	0.21 ± 0.15	0.50 ± 0.21
CDR	0.21 ± 0.25	0.50	0.47 ± 0.11	0.67 ± 0.24
GDS	2.00	2.70 ± 0.46	3.06 ± 0.40	3.50 ± 0.50

SCD, Subject Cognitive Decline; NaMCI Non-amnestic Mild Cognitive Impairment; aMCI, amnestic Mild Cognitive Impairment; AD, Alzheimer’s Disease; MMSE, Mini-Mental State Examination; SGDS, Short Form of Geriatric Depression Scale; B-ADL, Basic Activities of Daily Living; K-IADL, Korea-Instrumental Activities of Daily Living; CDR, Cognitive Dementia Rating; GDS, Global Deterioration Scale.

**Figure 2:**
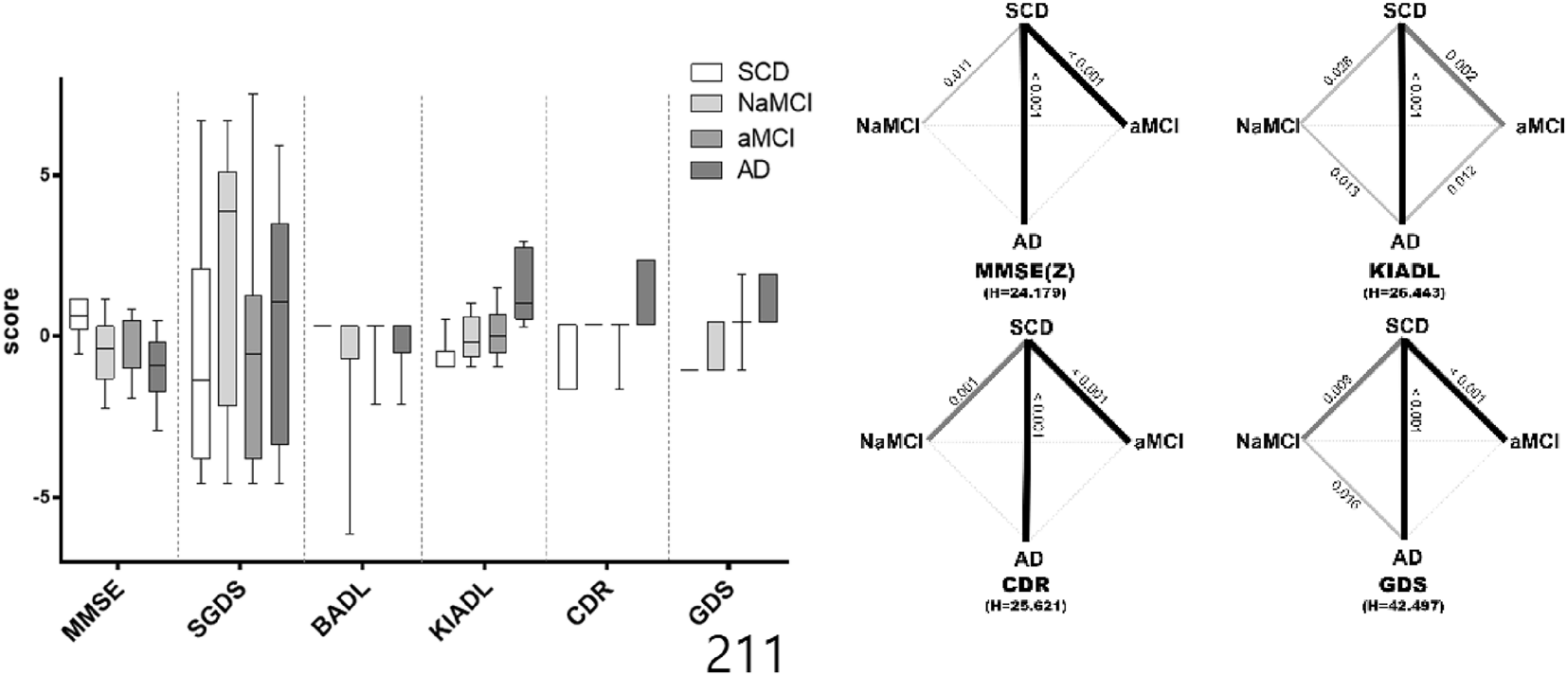
Statistical results of neurophysiological test: The left panel shows the scores according to each cognitive test, and the statistical values and results are shown using the *p *value in the right panel.

Numerical and continuous results were used for analysis. The participants labeled as SCD, naMCI, aMCI, and AD were patients at the Korea University Medicine. The SNSB-II was performed on the patients and/or caregivers with subjective memory complaints. CDR 0 was defined as SCD, CDR 0.5 as MCI, and CDR 1 as AD^29,30^. Compared to the age and education norms, MCI patients with in-memory domain scores below − 1.0 SD were classified as aMCI and otherwise, as naMCI^31,32^. Detailed values of the cognitive test scores for each group are presented in Table 2. To confirm the statistical difference in each group, a normality test was performed using the Shapiro–Wilk test, and normality was not found. Therefore, we performed the Kruskal–Wallis test, a non-parametric test, and post-hoc pairwise comparisons. The statistical differences between each group are shown in Fig. 2.

### Computer-based Cognitive Task

SNSB-II, a neuropsychological test, consists of eight cognitive measures to evaluate five cognitive domains: (1) Memory: the Seoul Verbal Learning Test (SVLT) delayed recall (verbal memory) and Rey-Osterrieth Complex Figure Test (RCFT) delayed recall (visual memory); (2) Language: Korean version of the Boston Naming Test (K-BNT); (3) Visuospatial function: RCFT copying Test; (4) Frontal executive function: tasks for animal names and supermarket items and a phonemic portion of the Controlled Oral Word Association Test (COWAT) and the Stroop Test (color reading); and (5) Attention: Digit Span Test forward and backward. This test using paper and pencil involves the examiner and patient facing each other. It takes about 30 min to 1 h, and there is a possibility of incorrect responses depending on the patient’s fatigue and tension. Accordingly, we proposed a computer-based cognitive task that unaffected by the examiner and is robust to the patient’s fatigue and tension. It consists of 11 tasks that match the 5 cognitive domains of existing neuropsychological tests. We conducted this task in a shorter timeframe, collected behavioral data on the subject’s correct response rate and reaction time, and simultaneously acquired EEG data during the cognitive process.

**Figure 3:**
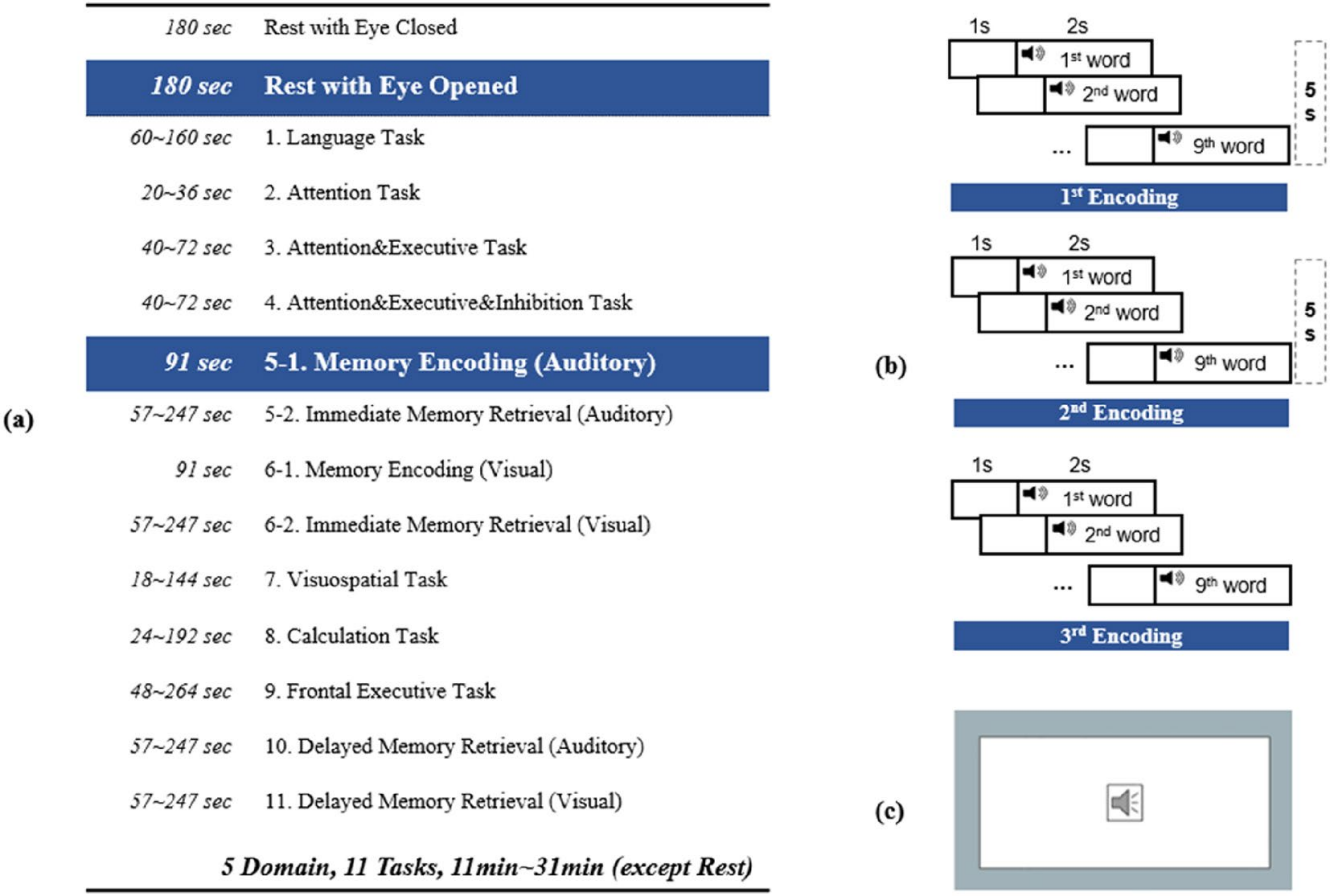
Experimental procedure. (**a**) List of subtests in the computer-based cognitive tasks; (**b**) Experimental procedure of memory encoding task (5–1 Subtest) and (**c**) Stimuli of encoding tasks.

Before starting the tasks, resting EEG data were obtained by asking the participants to rest with eyes closed, and with eyes opened, each for 3 min. Subsequently, EEG data were recorded during cognitive tasks. Cognitive tasks were assessed in five domains (Language, Attention, Frontal Executive, Memory, and Visuospatial). It consisted of 11 subtests and depending on response time, the total time ranged from 11 to 31 min. Based on the inputs from neurology experts at Korea University, this task was created using E-prime 3.0, a computerized cognitive test tool where event triggers and participants’ responses were recorded automatically to analyze behavioral responses and EEG. The participants’ responses were acquired using the Button key on the Serial Response Box (Chronos, Psychology Software Tools). The list of the subtest is reported in the Fig. 3a.

### Experimental procedures

This study, utilized EEG data measured during the “Memory Encoding (Auditory)” session of the computer-based cognitive task analysis. Memory is an important cognitive domain for diagnosing Alzheimer’s and is particularly important in distinguishing between naMCI and aMCI. Additionally, due to the nature of the paper and pencil test, the score was determined by asking about the memory results, and analysis of the memory process was not possible. However, by performing the proposed task, we were able to acquire EEG data while words were being remembered.

The instructions were explained to all participants and displayed on a 24-inch monitor, as shown Fig. 1 (right panel). This task consisted of three encoding sessions, each requiring 27 s, resulting in a total time of only approximately 91 s. As shown in Fig. 3b, at the beginning, participants focused on a white background for 1 s and listened to a word for 2 s (e.g., “Book,” “Table”, etc.). Nine words were auditorily presented (Fig. 3c) and the same words were repeated in the same order for each encoding session. The participants had a 5 s break in between each encoding session.

### Signal acquisition and processing

We used the ActiveTwo BioSemi system (Biosemi, Amsterdam, Netherlands) to acquire EEG signals when participants are at rest and performing memory-encoding tasks. Recordings were obtained from 64 scalp electrodes based on the 10/20 system, and the ground electrode during acquisition was formed by the Common Mode Sense active electrode and the Driven Right Leg passive electrode according to BioSemi’s design. The EEG signals were digitized using ActiView software (Biosemi) as 2048 Hz of the sampling rate and impedance was kept below 10 kΩ. MATLAB R2022b (Mathworks Inc., Natick, MA, USA) with the EEGLAB v2022.1 toolbox was used to analyze the EEG data. The data were downsampled to 512 Hz and bandpass-filtered with cutoffs of 2–100 Hz. To eliminate electrical line noise, an infinite impulse response notch filter at 60 Hz was used. An average reference was used and ICA was automatically applied to correct artifacts and noise caused by blinking, muscle movements, and electrocardiography. Encoding data were segmented for each trial, starting 500 ms before the onset of each stimulus and continuing for 2 s after. Baseline correction was applied with data obtained between − 500 and 0 ms of stimulus onset. To analyze EEG data obtained during the resting state, the middle 2-min interval was used for the analysis of data measured for 3 min with eye opened state.

### Feature extraction and selection

Feature extraction and selection were performed to obtain useful information from signals. For this purpose, we analyzed and extracted features that reflected the phase, spectral, and temporal characteristics during the rest and memory-encoding states. In phase analysis, features were obtained from three perspectives: total activity, phase-locked activity, and non-phase-locked activity^33^. Total activity refers to the time–frequency decomposition of each trial, and the time–frequency powers of all trials in each session were averaged, a commonly used time–frequency approach for cognitive electrophysiology studies. Phase-locked activity is phase-aligned with the time = 0 event; therefore, it is observed in time-domain and time–frequency-domain averaging. Non-phase-locked activity is the time–frequency representation of the data after the phase-locked components of the EEG signal are removed. One method of obtaining this activity is subtracting phase-locked activity from each trial and performing the single-trial time–frequency decomposition of the total power. We applied complex Morlet wavelets^34^ with frequencies ranging from 2 to 60 Hz that increased logarithmically to extract power and phase information. Because the total activity is composed of phase-locked and non-phase-locked activities, the non-phase-locked power is obtained by subtracting phase-locked activity from total activity.

In spectral analysis, absolute power (AP) and relative power (RP) are the most well-known indicators to measure power spectrum density of EEG^16,35,36^. AP is the square of the amplitude and RP is the percentage of the total power occupied by each frequency band. Power was calculated for the following frequency bands: 2–4 Hz (delta band), 4–7 Hz (theta band), 7–12 Hz (alpha band), 12–19 Hz (low beta band), 19–30 Hz (high beta band), and 30–40 Hz (gamma band). We divided temporal domain into 400 ms intervals, 400 ms before the start of the stimulus to 2000 ms after the start: − 400–0 ms, 0–400 ms, 400–800 ms, 800–1200 ms, 1200–1600 ms, and 1600–2000 ms. We obtained additional temporal features by subtracting the time epoch immediately before each interval.

We obtained 11 temporal features (six time epochs + five difference time epochs) in the encoding states and nine temporal features (five time epochs + four difference time epochs) in the resting state. In the encoding states, the data before stimulation influenced the encoding ability^37^; therefore, we included pre-stimulus data. In the resting state, because there was no stimulation, we used the data 0 ms. In^37^, a subset of electrodes was selected to reduce the dimensionality of a subsequent memory dataset. In our study, we followed these subset regions and showed the list of the selected electrodes in Table 3. Thus, the total number of features are *#brain regions *$\times$* #phase activity *$\times$* #power ratio *$\times$* #frequency bands *$\times$* #Time Epoch*. Subsequently, we extracted 2268 features from the resting state and 2772 features from each encoding state.

**Table 3:** Selected channel and their regions.

Regions	Selected electrode
FM (Frontal medial)	FPz, AFz, Fz, FP1, FP2
LT (Left temporal)	F5, F7, FC5, FT7, C5, T7
RT (Right temporal)	F6, F8, FC6, FT8, C6, T8
CM (Centro medial)	FCz, Cz, C1, C2, CPz
LP (Left posterior)	CP1, CP3, CP5, P3, P5
RP (Right posterior)	CP2, CP4, CP6, P4, P6
PM (Posterior medial)	Pz, P1, P2, POz

To prevent overfitting according to a large number of features, we selected features by applying two methods. First, we performed a one-way ANOVA for the four groups to select useful features from the significant extracted features, which had a *p *value of < 0.05. Therefore, 56 features in Resting, and 258 features in Encoding (84 features in 1st Encoding, 93 features in 2nd Encoding and 81 features in 3rd Encoding*)* were selected and used for classification. Afterwards, L2 regularization called Ridge Regression was performed. Ridge regression is a simple technique to reduce model complexity and prevent overfitting^52^. We attempted to prevent overfitting of the model by finding the coefficients of the ridge regression model and applying them as feature weights. Additionally, the complex architecture of deep neural networks is prone to overfitting due to their larger capacity to remember training data. To prevent this, an early stopping method was applied. If the validation loss did not improve for 5 consecutive epochs, training was stopped and the model was restored to the weight with the lowest validation loss.

### Classification

To address classification within the Alzheimer’s spectrum, we conducted a comprehensive analysis by employing and comparing prominent Machine Learning and Deep Learning models. In addition to conventional machine learning techniques, we sought to contribute to the forefront of research by implementing a Deep Learning model, a methodology gaining widespread utilization in recent studies^38–40^. MATLAB functions were used for the nine classification techniques; support vector machines with the 2-order polynomial kernel (*2SVM*) or 3-order polynomial kernel (*3SVM*), K-nearest neighbor with cosine distance metric (*cKNN*) or Euclidian distance metric (*EuKNN*), subspace Ensemble learning (*partEns*), neural network with narrow layer (*narNN*), wide layer (*wideNN*) or double layer (*doubleNN*), and *LDA*. The implementation of the three deep learning models, Dense Neural Network (DNN), Multilayer Perceptron(MLP) and 1D-Convolution Neural Network (1D-CNN), was performed in Python 3.9.13, Tensorflow 2.13.0, and Keras 2.13.1. The DNN model consisted of three layers of 128-64-32 with relu activation functions. We include a dropout layer to reduce overfitting with a dropout ratio of 0.5. The MLP model consisted of two layers with kernel sizes of 256 and 128 with relu activation functions. The 1D-CNN model consisted of 64 × 3 Conv1D layers with relu activation function and used the Relu activation function. Additionally, the size of the feature map was reduced using Maxpoolling1D layer with a window size of 2, and a flatten layer and dense layer were applied. The output layer of all models used softmax activation to return classes with probabilities for each class as the model’s predictions. These models were applied to the four-class classification of SCD, naMCI, aMCI, and AD. To evaluate the performance, we used the LOSO strategy^1,21^. This study used *sensitivity, specificity, precision, F1-score*, *accuracy*, and *area under of receiver operating characteristic curve (AUC)* to evaluate the model’s performance. These criteria are presented in Eqs. (1)–(5) using the labels true positive (TP), true negative (TN), false positive (FP), and false negative (FN). Sensitivity assesses the model’s capacity to accurately identify true positives among the actual positives (such as patients). The low sensitivity scores of the model indicate that some positive patients are incorrectly labeled as negatives. Specificity measures the model’s ability to correctly predict the negatives out of actual negatives. It is similar to sensitivity but focuses on “False,” not True. Precision evaluates the ratio of accurately predicted positive labels out of all the positive predictions made. The low precision scores indicate the misclassification of certain actual negative cases as positive. F1 score serves as a comprehensive metric, combining the precision and sensitivity scores through a harmonic mean, to offer valuable insights into the overall quality of the model’s output. Meanwhile, Accuracy is calculated by comparing the true positives and true negatives with all the positive and negative observations.

$$sensitivity = \frac{TP}{{TP + FN}} \times 100\%$$

$$specificity = \frac{TN}{{TN + FP}} \times 100\%$$

$$precision = \frac{TP}{{TP + FP}} \times 100\%$$

$$F1\,score = 2 \times \frac{precision \times sensitivity}{{precision + sensitivity}} \times 100\%$$

$$accuracy = \frac{TP + TN}{{TP + TN + FP + FN}} \times 100\%$$

### Ethics approval and consent to participate

Study approval by the Institutional Review Board of Anam Hospital, Korea University was obtained before the study initiation. All participants consented to participate in this study, which was conducted in accordance with the Declaration of Helsinki. (No. 2021AN0266).

## Results

### Results of correlation between neuropsychological test score and computer-based cognitive task performance

**Figure 4:**
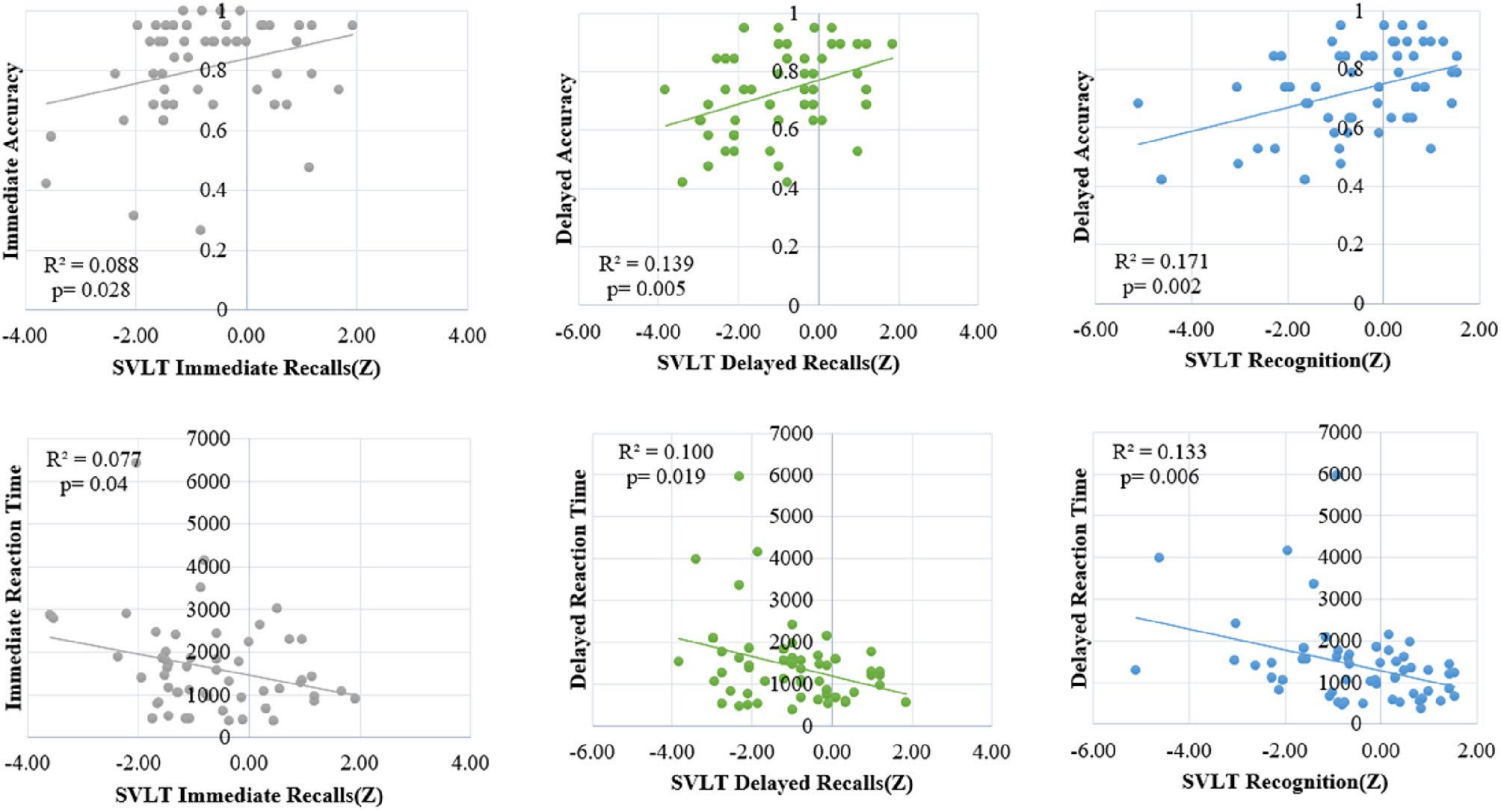
Correlation between test score of memory domain in SNSB and performance (accuracy and reaction time) of memory retrieval in computer-based cognitive task.

We analyzed the correlation between the score of neuropsychological test and the performance (accuracy and reaction time) of the computer-based cognitive cask to assess the reliability of this task in classifying groups. As a result, a significant correlation (*p* < 0.05) was found in all five cognitive domains (“Appendix Table”). Among the 11 subtests we presented, this study focused on the memory encoding session. Immediate memory retrieval were matched with SVLT immediate and delayed memory retrieval, indicating the memory encoding task concentration, were matched with SVLT delayed, and SVLT Recognition scores. As a result, as shown in Fig. 4, it was confirmed that in all matches, the higher the SVLT score, the higher the accuracy and the longer the reaction time.

**Figure 5:**
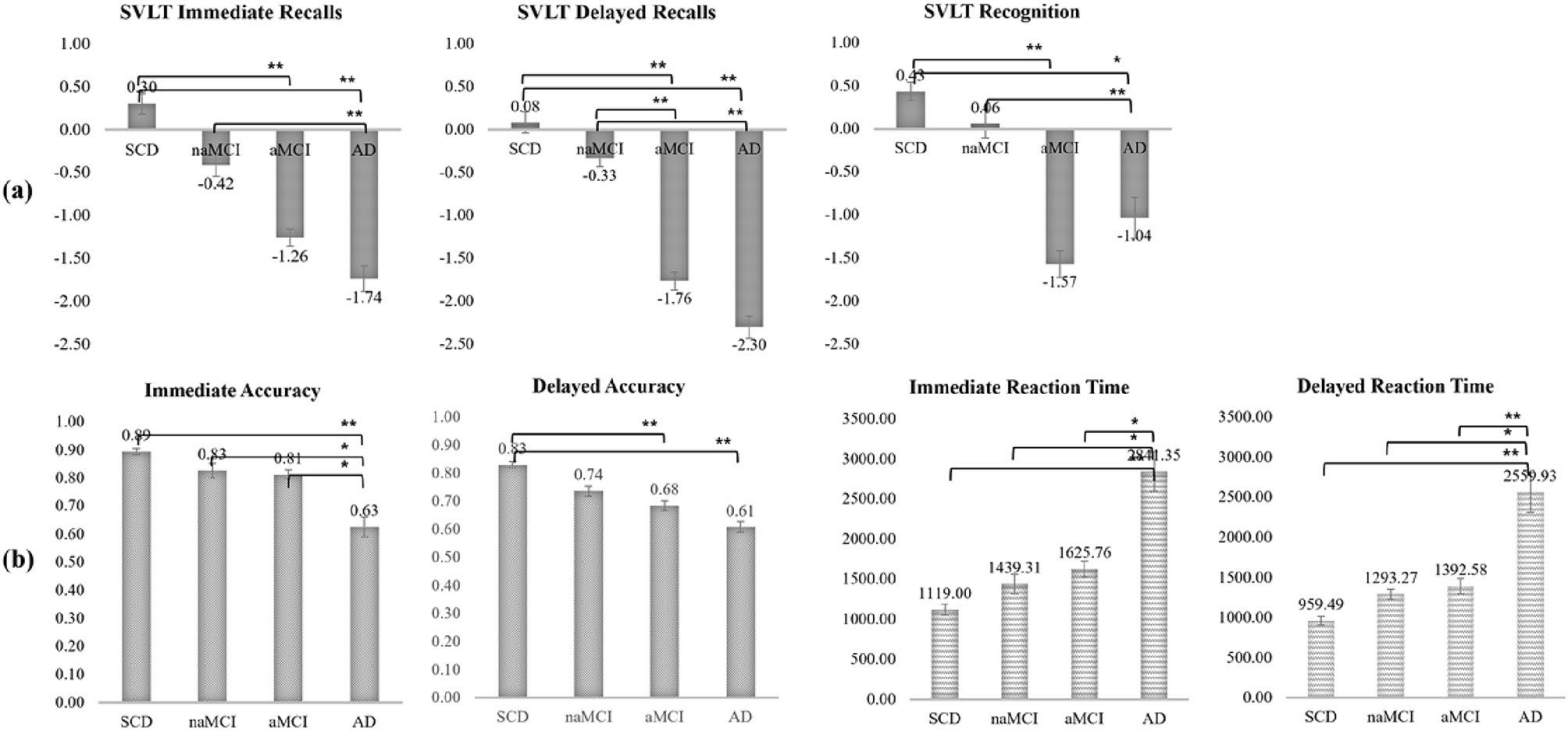
Comparison of the memory score of each group (**a**) The results of SNSB-II Test (SVLT immediate, SVLT delayed and SVLT recognition); (**b**) The results of performance (accuracy and reaction time) according to Computer-based cognitive task (immediate and delayed retrieval).

As show in Fig. 5a, SVLT immediate recall scores were significantly difference between each group (SCD vs. aMCI, *p* = 0.00003; SCD vs. AD, *p* = 0.00001; naMCI vs. AD, *p* = 0.015). SVLT delayed recall scores also had significant differences between each group (SCD vs. aMCI, *p* = 0.000002; SCD vs. AD, *p* = 0.000001; naMCI vs. aMCI, *p* = 0.0018; naMCI vs. AD, *p* = 0.0002). Additionally, SVLT recognition score had significant difference between each group (SCD vs. aMCI, *p* = 0.00006; SCD vs. AD, *p* = 0.025; naMCI vs. aMCI, *p* = 0.008). However, in the SVLT test, no difference was found between SCD and naMCI, or between aMCI and AD.

The comparison of each group’s performance of memory retrieval is shown in Fig. 5b. The accuracy of immediate memory retrieval had significant difference between each group (SCD vs. AD, *p* = 0.0003; naMCI vs. AD, *p* = 0.025; aMCI vs. AD, *p* = 0.017) and the same is true for delayed memory retrieval (SCD vs. aMCI, *p* = 0.005; SCD vs. AD, *p* = 0.0003). In terms of immediate and delayed memory retrieval, reaction time also showed significant differences between each group (SCD vs. AD, *p* = 0.0002 in immediate and delayed; naMCI vs. AD, *p* = 0.01 in immediate and delayed; aMCI vs. AD, *p* = 0.012 in immediate and *p* = 0.009 in delayed). In the memory retrieval task, AD showed significant differences from all groups, but no significant differences were found among the other groups. However, a gradual decline in accuracy and an increase in reaction time were confirmed for each group. These results demonstrated the reliability of the memory encoding task performed by our computer-based cognitive task in measuring cognitive ability.

### Comparison of selected features in each session

Table 4 presents the number of selected features in each session. In the *Resting state*, for the brain regions, frontal medial features were selected frequently. Phase activity & power ratio favored induced absolute power, and Alpha was the most frequently chosen frequency band. In the encoding state, the *2nd Encoding* had the highest feature selection, followed by the *1st and 3rd encoding*. Right Temporal, Central medial and parietal medial contributed the most features in the overall encoding process in terms of brain regions. More specifically, right temporal in *1st Encoding*, parietal medial, central medial, and left temporal in *2nd Encoding*, and parietal medial, left parietal, and right parietal in *3rd Encoding* were selected as characteristics to distinguish the four groups. For the Phase activity & Power ratio, in the rest state, induced absolute power had the most selected features in the *2nd* and *3rd Encoding,* while total relative power had numerous features selected in the *1st Encoding*. For the frequency band, Delta was prominent in *1st Encoding*, Beta2 and Gamma in *2nd Encoding*, and Beta2 in *3rd Encoding*. Finally, in the characteristics of the time epoch, numerous features appeared immediately after hearing the word (8to12 and 12to16) in the *1st Encoding*, at the beginning of the word (0to4) and immediately after hearing it (12to16) in the *2nd Encoding*, and before listening to the word in *3rd Encoding*.

**Table 4:** The number of selected features in Resting and Encoding states.

Feature list		Resting with eye opened	*1st encoding*	*2nd encoding*	*3rd encoding*
The total number of features		***56***	***84***	***93***	***81***
Brain regions	FM	**18**	11	0	5
	LT	5	12	17	6
	RT	6	**24**	14	10
	CM	1	14	17	13
	PM	6	14	**18**	**17**
	LPS	10	2	13	15
	RPS	10	7	14	15
Phase activity & Power ratio	Total_A	11	15	20	13
	Induced_A	**23**	14	**37**	**19**
	Eovked_A	4	14	17	9
	Total_R	5	**20**	10	12
	Induced_R	1	16	5	17
	Evoked_R	12	5	4	11
Frequency band	DELTA	4	**21**	11	14
	THETA	12	14	9	11
	ALPHA	**15**	11	10	15
	BETA1	10	9	19	7
	BETA2	10	17	**23**	**22**
	GAMMA	5	12	21	12
Time Epoch	EPOCH				
*(a)*	pre4	–	9	3	**15**
*(b)*	0to4	2	3	**10**	3
	4to8	7	5	3	0
*(c)*	8to12	7	**12**	7	9
*(d)*	12to16	8	**12**	**10**	8
	16to20	5	8	5	7
	DIFF				
*(b)–(a)*	0to4-pre4	–	3	11	4
*(b)*	4to8-0to4	10	**11**	10	5
*(c)–(b)*	8to12-4to8	3	3	5	10
*(d)–(c)*	12to16-8to12	4	**11**	13	**14**
*(d)*	16to20-12to16	10	7	**16**	6

FM, Frontal Medial; LT, Left Temporal; RT, Right Temporal; CM, Central Medial; PM, Parietal Medial; LP, Left Posterial; RPP, Right Posterior; Total_A, Total Absolute Power; Induced_A, Induced Absolute Power; Evoked_A, Evoked Power; Total_R, Total Relative Power; Induced R, Induced Relative Power; Evoked_R, Evoked Relative Power.

(a): before word onset.

(b): during word sound.

(c): after word sound.

(d): review the word yourself.

### Classification results of all models in resting and encoding states

**Figure 6:**
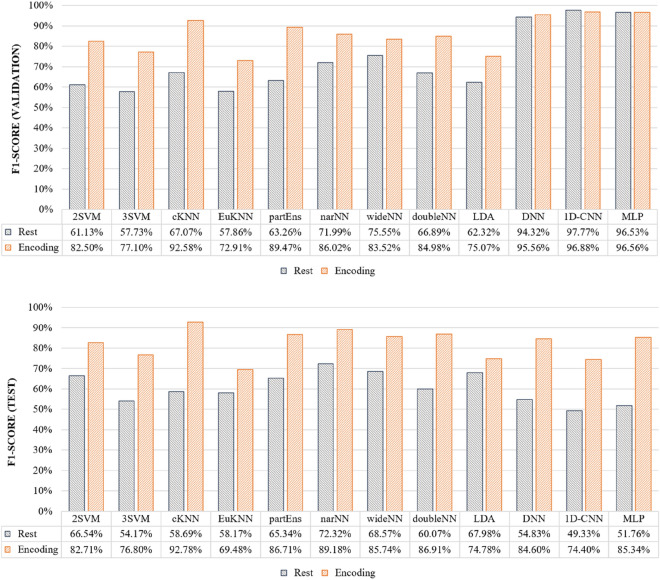
Comparison of classification results (F1 score) between EEG feature in resting and encoding states.

We classified SCD, naMCI, aMCI, and AD simultaneously for resting and encoding states. For the encoding state, all the features selected in each encoding session (*1st Encoding, 2nd Encoding* and *3rd Encoding*) were used. Figure 6 shows the validation and test results of the four classes classified using the nine ML models and three DL models. In both validation and test results, using the memory encoding state yielded higher F1 scores for all models compared to the rest state. On the model side, in the case of validation results, DL models (DNN, 1D-CNN and MLP) showed higher F1-scores than ML models overall, but in test results, narNN achieved the highest F1 score at 73.23% in the resting state, and cKNN achieved the highest F1 scoare at 92.78% in the encoding state.

### Comparing the classification results between resting and encoding states among the four groups

**Figure 7:**
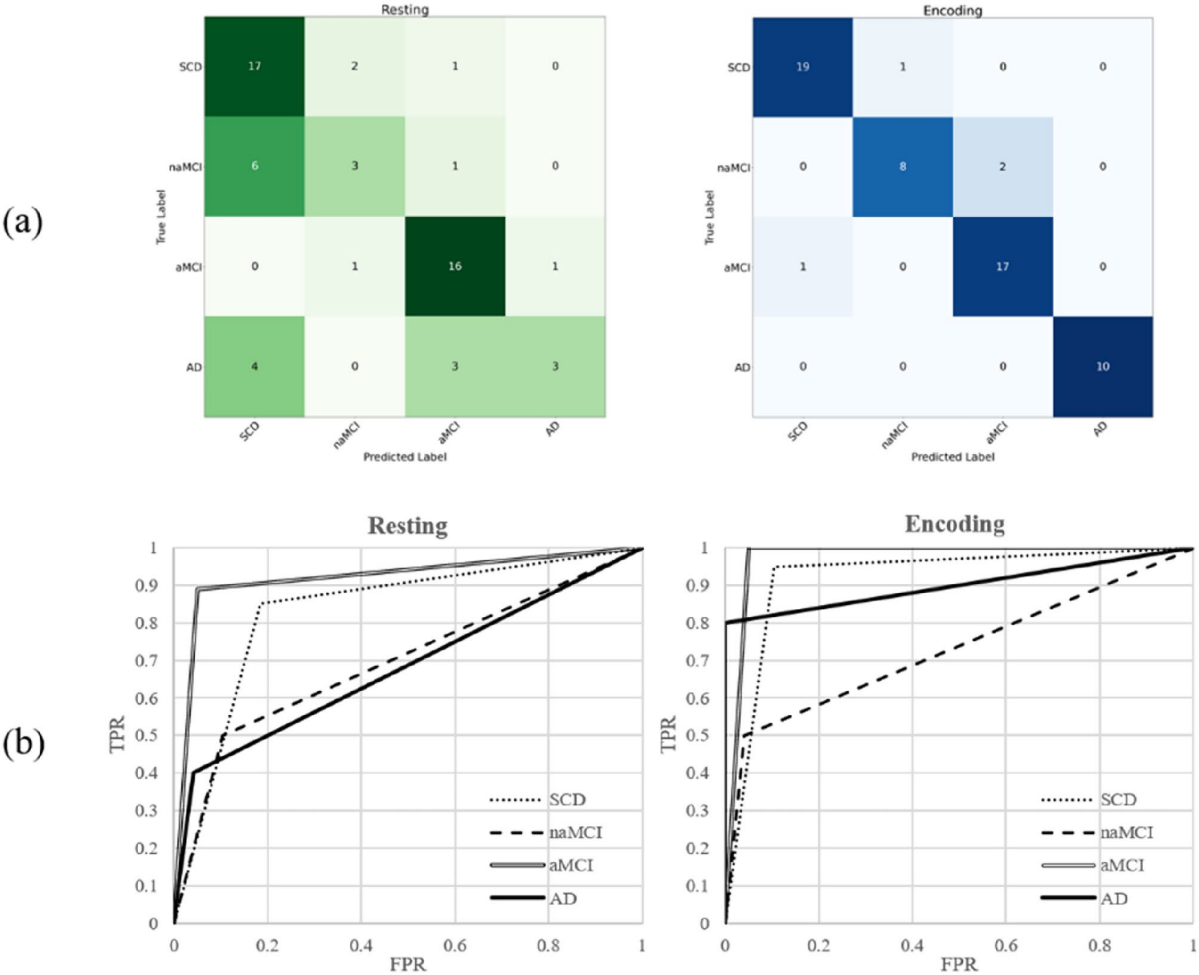
Performance evaluation using cKNN in resting and encoding state (**a**) Confusion Matrix; (**b**) receiver operating characteristic (ROC) curve.

Thus cKNN emerged as the best model with the highest F1 score, the test performance of each group was compared more detailly using sensitivity, specificity, precision, F1 score, and AUC. As shown in Table 5, in the Encoding states, all groups exhibited better performance compared to the resting state. The overall accuracy was higher in encoding states (93.10%) than in resting states (67.24%). Moreover, the Kappa score was 0.816, demonstrating the best model reliability in encoding states. The low F1 score the resting state was because of the low sensitivity values of naMCI (sensitivity: 30%) and AD (sensitivity: 30%) compared to SCD (sensitivity: 85%) and aMCI (sensitivity: 88.89%). However, in the encoding state, all groups had high sensitivity and overall high performance. The AUC value for the four groups were 0.962, 0.890, 0.947 and 1, respectively. Figure 7a shows the confusion matrix for the four groups classified using the cKNN for the resting and encoding states. Each true class is displayed in a row and each predicted class is displayed in a column. Figure 7b presents the receiver operating characteristic (ROC) curve of the cKNN model, with the x-axis representing FP rate and the y-axis representing TP rate. The AUC of each group was obtained through the area under the ROC curve.

**Table 5:** Classification results of the four groups in resting and encoding states using cKNN model.

	Resting	Encoding
		SCD
Sensitivity	85.00%	95.00%
Specificity	73.68%	97.37%
Precision	62.96%	95.00%
F1 score	72.34%	95.00%
AUC	0.793	0.962
		naMCI
Sensitivity	30.00%	80.00%
Specificity	93.75%	97.92%
Precision	50.00%	88.89%
F1 score	37.50%	84.21%
AUC	0.619	0.890
		aMCI
Sensitivity	88.89%	94.44%
Specificity	87.50%	95.00%
Precision	76.19%	89.47%
F1 score	82.05%	91.89%
AUC	0.882	0.947
		AD
Sensitivity	30.00%	100.00%
Specificity	97.92%	100.00%
Precision	75.00%	100.00%
F1 score	42.86%	100.00%
AUC	0.640	1
Accuracy	67.24%	93.10%
Kappa	0.126	0.816

### Evaluation of the overfitting in our result

**Figure 8:**
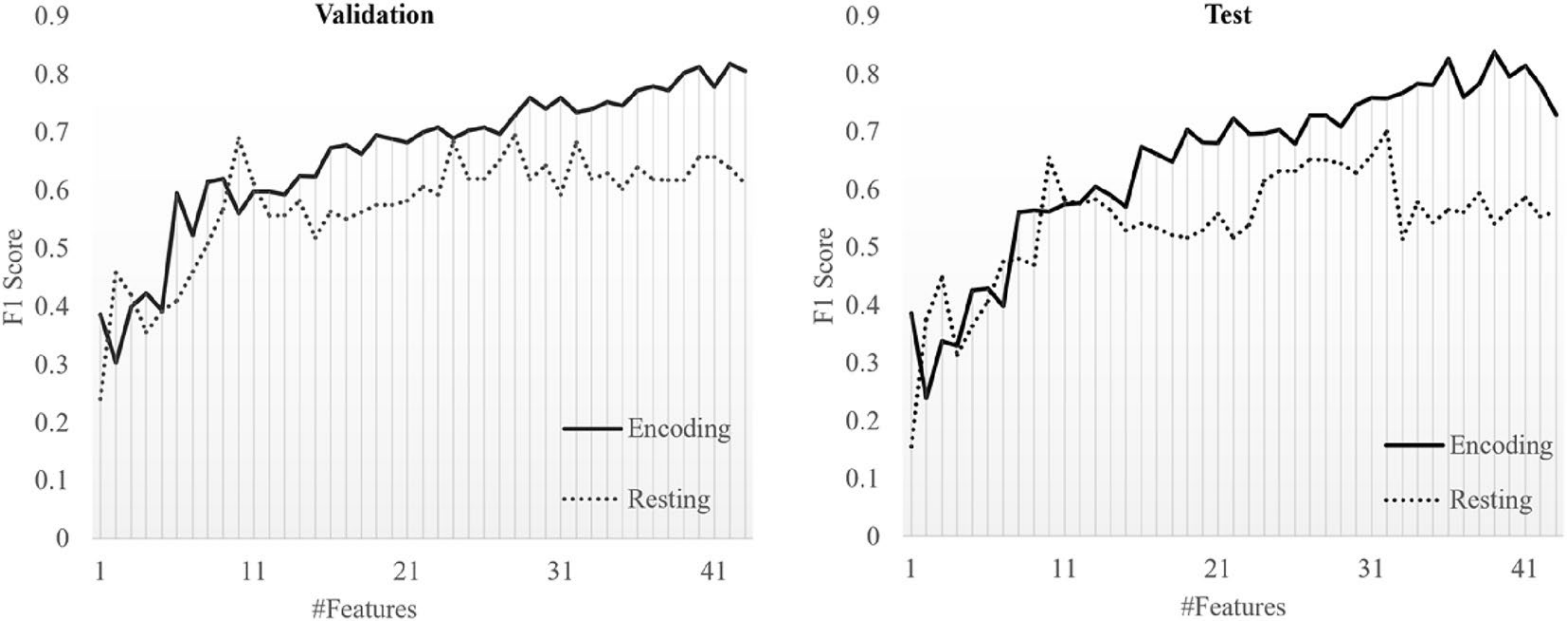
F1 score of performance results according to the number of features in resting and encoding states using cKNN.

In our research, we applied the LOSO strategy to evaluate the model across multiple sub-datasets, aiming to prevent overfitting. Additionally, we employed Ridge regularization to constrain the magnitude of weights, enhancing the model’s stability. Moreover, in machine learning, the number of features can affect accuracy. Therefore, we analyzed the F1-score according to the number of features for resting and encoding states. The features were sorted based on the p value of the ANOVA applied as the first feature selection method (see in “Feature extraction and selection” section), and the feature with the lowest *p* value was used for classification. The results confirmed that *Encoding state* had better classification accuracies, even with the same number of features as *Resting state* (Fig. 8).

## Discussion

While numerous studies focus on developing biomarkers for MCI to detect AD pathology early, distinguishing SCD from MCI, particularly naMCI, remains a challenge. To the best of our knowledge, this is the first study to classify SCD, naMCI, aMCI, and AD simultaneously. Additionally, we have also developed a computer-based cognitive task that outperforms the conventional time-consuming and laborious neuropsychological tests such as SNSB-II. We validated the proposed method by demonstrating a significant correlation between its performance and the scores of neuropsychological tests of clinical standard. We found that, all nine applied ML models achieved a more accurate classification of the AD spectrum using the EEG data acquired during the cognitive tasks as features compared to using data from the resting-stat.

The proposed computer-based task can measure cognitive areas similar to existing neuropsychological tests. It is time-efficient and provides robust results using biometric information to prevent measurement discrepancies owing to subject tension or mistake. The performance of this task, which consists of 11 subsets for 5 domains, showed significant correlation with the score of the neuropsychological test. We focused on memory, a representative cognitive impairment domain in AD. A significant correlation was confirmed between the memory score of the neuropsychological test and the accuracy and reaction time of the computer-based cognitive task, and a clear difference in performance between the four groups was confirmed. Accordingly, we were able to determine the effectiveness of memory encoding process in this task and validate the reliability of analyzing EEG data during the memory encoding process. Particularly, existing neuropsychological tests assess memory through responses to memory recall, but we were able to analyze EEG data during the memory encoding process through the proposed method.

Encoding is the initial memory process in which information that enters the brain through sense organs is learned and remembered^41–43^. Encoding stores information, but a post-encoding process called consolidation must be performed to continuously maintain and store this information as a more solid memory. Memory retention is difficult if they are not well solidified. Existing tests determine memory capacity through retrieval after storage. Particularly, because the SCD and naMCI groups had normal memory by definition, it was difficult to clinically distinguish them from the normal group in the memory domain. However, we were able to measure the EEG data during memory registration and encoding through the proposed computer-based cognitive task. Hence, through this classification, distinguishing features of SCD, naMCI, aMCI and AD were confirmed, resulting in a high performance of 93.10% when classifying these four groups. This is an encouraging result for early diagnosis because it helps differentiate SCD and naMCI, which have no memory decline in clinical diagnosis, when comparing brain activity during memory encoding. Particularly, EEG data during memory encoding showed higher performance in Alzheimer’s disease spectrum classification across all models both ML and DL, indicating a uniform result without model bias. In the cKNN model, which demonstrated the best performance, utilizing features selected during the resting state resulted in relatively favorable F1-scores for classifying the SCD group (72.34%) and aMCI group (82.05%). However, the classification performance was notably lower for the naMCI group (37.50%) and AD group (42.86%). However, using features selected during encoding for classification resulted in high F1 scores for SCD, naMCI, aMCI and AD as 95%, 84.21%, 91.89% and 100%, respectively. Additionally, using only the top 20 features instead of all features led to the F1 score exceeding 70%. This means that the higher classification performance in the memory encoding state is due to the characteristics themselves and not the number of features.

Studying the features selected according to the number of repetitions of encoding revealed interesting outcomes. Previous studies, have indicated memory-related areas to be included in the parietal medial and left temporal regions^18,44,45^. Consequently, during the overall memory encoding process, the area with the most selected features was the parietal medial. Interestingly, in Encoding 1, where the first word is presented, numerous features were selected from Right Temporal. A possible explanation for this is that, despite LT being used, the relevant area is severely damaged in AD and aMCI, that even RT is used. Therefore, the RT features was often used to distinguish SCD, naMCI, aMCI, and AD, and these features would have been further characterized in the 1st encoding when hearing the word for the first time. This approach of interpreting the neuropathological abnormalities of MCI/mild AD as a compensation has been discussed in other studies^46–51^. Also, in terms of time, during the first encoding process, many features were selected immediately after hearing the word, and during the second listening, they were selected immediately after the word began and immediately after the word ended. And finally, in the third listening, many features were selected from the data before hearing the word. However, we know that surface-level EEG data do not allow us to draw any conclusions about the underlying neural generators. This means that our interpretation mentioned above are some factors that influenced classification accuracy and does not imply that it is related to neural activity^53^. Considering these results, we intend to further investigate the point of focus depending on the number of repetitions during memory encoding by applying the high temporal resolution characteristics of EEG in our next study.

This study is the first proposing a 4-type classification including SCD and naMCI within the Alzheimer’s spectrum by applying both ML and DL. Nevertheless, our study is subject to certain limitations, including a limited number of samples and the exclusive use of data collected from a single hospital without external comparisons. Consequently, the performance of DL models in our tests was observed to be inferior to that of cKNN, a relatively straightforward model. We posit that with a more extensive dataset, our findings can be more effectively applied to enhance the performance of DL models. It is crucial to note that the discussion regarding the aforementioned limitations is based solely on the interpretation of the results obtained in this study, signifying a potential avenue for future research to gain a deeper understanding.

## Conclusion

In terms of Memory Domain, the main cognitive impairment in Alzheimer’s, the early diagnosis of SCD and naMCI, which are clinically normal but distinct from healthy controls, is crucial for the early diagnosis and prediction of Alzheimer’s. Therefore, this study aimed at classifying the subjects into four groups, SCD, naMCI, aMCI and AD, by applying various ML and DL models based on the EEG data acquired during the resting state and memory encoding state. Through this, we suggested that brain waves during actual cognitive processes are more meaningful in diagnosing the Alzheimer’s disease spectrum. Moreover, the cKNN model used for classification achieved an accuracy of 93.10% using the EEG data during memory encoding. Additionally, the computer-based cognitive task proposed in this study is time-effective compared to the existing neuropsychological tests as it measures five cognitive areas within 30 min and it can also minimize the bias depending on the condition of the examiner and subject by simultaneously measuring the patient’s behavioral data and biometric data. Performance results of this task showed a significant correlation with the scores obtained through neuropsychological tests, indicating the effectiveness of this test in measuring cognitive impairments and its applicability in complementing existing neuropsychological tests.

### Supplementary Information

Supplementary Information.

## Data Availability

The datasets used and/or analysed during the current study are available from the corresponding author on reasonable request.

## Abbreviations

AD: Alzheimer’s disease
MCI: Mild cognitive impairment
naMCI: Non-amnestic MCI
aMCI: Amnestic MCI
SCD: Subjective cognitive decline
EEG: Electroencephalography
CSF: Cerebrospinal fluid
ATN: The Amyloid/Tau/Neurodegeneration
PET: Positron emission tomography
HC: Health control
LDA: Linear discriminant analysis
PSD: Power spectral density
CST: Continuous wavelet transform
BiS: Bispectral
EC: Eyes-closed resting state
EO: Eyes-opened resting state
MST: Motor speed test
LLC: Log linear connectivity,
MLR: Multinomial and logistic regression
MLP: Multi layer perceptron
RP: Relative power
AP: Absolute power
DWT: Discrete wavelet transform,
FTT: Finger tapping test
CPT: Continuous performance test
VaD: Vascular dementia
ML: Machine learning
DL: Deep learning
MMSE: Mini-mental state examination
SNSB-II: Seoul neuropsychological screening battery second edition
SGDS: Short form of geriatric depression scale
B-ADL: Basic activities of daily living
K-IADL: Korea-instrumental activities of daily living
CDR: Cognitive dementia rating
GDS: Global deterioration scale
SVLT: Seoul verbal learning test
RCFT: Rey-Osterrieth complex figure test
K-BNT: Korean version of the Boston Naming Test
COWAT: Controlled oral word association test
FM: Frontal medial
LT: Left temporal
RT: Right temporal
CM: Centro medial
LP: Left posterior
RP: Right posterior
PM: Posterior medial
2SVM: The 2-order polynomial kernel
3SVM: 3-Order polynomial kernel
cKNN: K-nearest neighbor with cosine distance metric
EuKNN: Euclidian distance metric
partEns: Subspace Ensemble learning
narNN: Neural network with narrow layer
widen: Wide layer
doubleNN: Double layer
DNN: Dense neural network
MLP: Multilayer perceptron
1D-CNN: 1D-convolutional neural network
AUC: Area under of receiver operating characteristic curve
TP: True positive
TN: True negative
FP: False positive
FN: False negative
