# Supplementary material for: Electroencephalography-based classification of Alzheimer’s disease spectrum during computer-based cognitive testing
Source: Sci Rep. 2024 Mar 4;14:5252. doi: 10.1038/s41598-024-55656-8 (PMC10912091; doi:10.1038/s41598-024-55656-8)
Supplement: Supplementary file 1 — Supplementary Information. [file 41598_2024_55656_MOESM1_ESM.docx]

**Appendix**

Appendix A.1 Correlation between Neuropsychological Test(SNSB) and Computer-based Cognitive Task in 5 Cognitive Domains.

| **Cognitive Domain** | **Neuropsychological Test** | **Computer based Cognitive Task** | **r** | **p** |
| --- | --- | --- | --- | --- |
| **Memory** | SVLT_imeediate_recalls(Z) | Immediate ACC | 0.296 | 0.028 |
|  |  | Immediate RT | -0.278 | 0.040 |
|  |  | Delayed ACC | 0.414 | 0.002 |
|  |  | Delayed RT | -0.280 | 0.038 |
|  |  | Delayed stdRT | -0.286 | 0.034 |
|  | SVLT_delayed_recalls(Z) | Immediate RT | -0.311 | 0.021 |
|  |  | Delayed ACC | 0.372 | 0.005 |
|  |  | Delayed corRT | -0.272 | 0.045 |
|  |  | Delayed RT | -0.316 | 0.019 |
|  | SVLT_Recognition(Z) | Delayed ACC | 0.431 | 0.002 |
|  |  | Delayed corRT | -0.359 | 0.007 |
|  |  | Delayed RT | -0.365 | 0.006 |
|  |  | Delayed stdRT | -0.302 | 0.025 |
|  | RCFT_immediate_recall(Z) | Immediate ACC | 0.485 | 0.000 |
|  |  | Immediate RT | -0.304 | 0.024 |
|  |  | Immediate corRT | -0.274 | 0.043 |
|  |  | Immediate stdRT | -0.362 | 0.007 |
|  |  | Delayed ACC | 0.439 | 0.001 |
|  | RCFT_delayed_recall(Z) | Immediate ACC | 0.518 | 0.000 |
|  |  | Immediate RT | -0.314 | 0.019 |
|  |  | Immediate corRT | -0.299 | 0.027 |
|  |  | Immediate stdRT | -0.374 | 0.005 |
|  |  | Delayed ACC | 0.499 | 0.000 |
|  | RCFT_Recognition(Z) | Immediate ACC | 0.380 | 0.004 |
|  |  | Immediate RT | -0.300 | 0.026 |
| **Attention** | DST_F+B(Z) | Go100 stdRT | -0.266 | 0.048 |
|  | DST_Forward(Z) | Go500 RT | -0.270 | 0.044 |
|  |  | Go500 corRT | -0.387 | 0.003 |
| **Language** | Repetition | Math RT | -0.306 | 0.022 |
|  |  | Math falRT | -0.274 | 0.041 |
|  | Calculation | Math acc | 0.121 | 0.002 |
|  |  | Math RT | -0.164 | 0.007 |
|  |  | Math corRT | -0.117 | 0.006 |
|  |  | Math stdRT | -0.290 | 0.000 |
|  | Praxis_ideomotor | Math falRT | -0.238 | 0.039 |
|  |  | Math stdRT | -0.555 | 0.030 |
| **VisuoSpatial** | CDT | Line Acc | 0.460 | 0.000 |
|  | RCFT_score(Z) | Line Acc | 0.275 | 0.040 |
|  |  | Line falRT | -0.328 | 0.014 |
|  |  | Line RT | -0.318 | 0.017 |
|  |  | Line stdRT | -0.299 | 0.025 |
|  | RCFT_time(Z) | Line corRT | -0.294 | 0.028 |
|  |  | Line falRT | -0.351 | 0.008 |
|  |  | Line RT | -0.347 | 0.009 |
|  |  | Line stdRT | -0.306 | 0.022 |
| **Frontal** | COWAT_Animal(Z) | Stroop falRT | -0.419 | 0.001 |
|  |  | Stroop RT | -0.273 | 0.042 |
|  | COWAT_Supermarket(Z) | Stroop falRT | -0.328 | 0.014 |
|  | COWAT_Phonemic_total(Z) | Stroop falRT | -0.310 | 0.020 |
|  | COWAT_Animal+ㄱ(Z) | Stroop falRT | -0.409 | 0.002 |
|  | CWST_CR_no.of_correct_response(Z) | Stroop corRT | -0.301 | 0.024 |
|  |  | Stroop falRT | -0.341 | 0.010 |
|  |  | Stroop RT | -0.295 | 0.027 |
|  |  | Stroop Rtvar | -0.301 | 0.024 |
|  | CWST-60_CR_correct(Z) | Stroop falRT | -0.334 | 0.012 |
|  |  | Stroop Rtvar | -0.334 | 0.012 |
|  | TMT_partA(Z) | Stroop corRT | -0.384 | 0.004 |
|  |  | Stroop falRT | -0.350 | 0.008 |
|  |  | Stroop RT | -0.381 | 0.004 |
|  |  | Stroop Rtvar | -0.291 | 0.029 |
|  | TMT_partB(Z) | Stroop falRT | -0.275 | 0.040 |
